# Impostor phenomenon and its association with resilience in medical education – a questionnaire study among Swedish medical students

**DOI:** 10.1186/s12909-024-05788-2

**Published:** 2024-07-19

**Authors:** Emelie Kristoffersson, Jens Boman, Aziz Bitar

**Affiliations:** Department of Clinical Science, Professional Development, Umeå University, Umeå, 901 87 Sweden

**Keywords:** Impostor phenomenon, Clance Impostor Phenomenon Scale; resilience, Brief resilience scale, Questionnaire, Undergraduate medical students

## Abstract

**Background:**

Concern over medical students’ well-being is a global issue, with studies showing high psychological distress rates. Impostor Phenomenon (IP), i.e., underestimating one’s abilities, attributing success to external factors, and feeling like a fraud, has been implicated as one reason behind these troubling findings. Meanwhile, resilience has been suggested to protect against psychological distress. This study aimed to investigate the prevalence of IP and its association with resilience among undergraduate medical students.

**Methods:**

The Clance Impostor Phenomenon Scale (CIPS), the Brief Resilience Scale (BRS), and sociodemographic questions were completed by 457 medical students registered in their 2-10th semester at a Swedish university. Of the respondents, 62.6% identified as women, 36.1% as men, and 1.3% as others.

**Results:**

The prevalence of IP was 58.4% (defined as CIPS score *≥* 62). According to the CIPS scoring guidelines, 10.3% of participants had low IP, 29.5% moderate, 41.6% frequent, and 18.6% intense IP. Of all participants, almost 90% experienced at least moderate and 60.2% frequent to intense IP. Women had significantly higher CIPS scores and lower BRS scores than men. In contrast, neither attending semester nor age group significantly impacted CIPS scores. Finally, there was a moderate inverse correlation between the level of resilience and the prevalence of IP.

**Conclusions:**

Our findings suggest that the majority of Swedish medical students feels like an impostor, and of these students, most do so frequently. Furthermore, IP is more prevalent among female students – who also show lower levels of resilience. Moreover, our results indicated that IP could hinder achieving higher resilience. Future longitudinal studies should investigate how gender norms may contribute to IP feelings and explore the potential advantages of counteracting IP and strengthening resilience among medical students. However, targeted interventions addressing individual students’ IP and resilience are insufficient. There is also a need to address structural aspects of the educational environment, such as gender stereotypes, that may contribute to IP.

## Background

The well-being of medical students is cause for concern within higher education worldwide, with studies showing that they – especially women – display high levels of mental illness [1–5]. Thus, it becomes increasingly clear that medical schools must proactively support students’ well-being and learn more about both risk factors and protective factors.

Studies indicate that medical students have higher levels of stress [6, 7], depression, anxiety, burnout, suicide, and suicidal ideation than the general population [1, 2, 4, 5]. Furthermore, there is reason to believe that mental health issues commencing in medical school continue as students start working [8, 9]. Many proposed reasons exist for the high mental health burden within the medical student population. The excessive amounts of stress, high expectations, competitiveness, perfectionism, and clinical practice characterized by high workloads and anxiety about making mistakes have all been suggested to put students in medical school at risk for psychological distress [2, 10, 11]. Additionally, the competitive environment puts high-achieving students at risk of developing IP – implicated as a strong predictor of psychological distress [12, 13].

The IP or impostor experience was initially described by Clance and Imes in 1978 among high-achieving women. The term refers to high-achieving individuals who inaccurately believe they have fooled others into thinking they are more capable than they are, attribute their success to external causes, and live with chronic insecurity and fear of being unmasked as a fraud [14]. IP is known to be more prevalent in the medical community than other fields, possibly because high-performers and perfectionist are over-represented in this group [12, 13, 15] and because its demanding nature with low acceptance of failure, high responsibilities, and competitive environment means that IP-feelings are at risk of being amplified [12, 13]. Among medical students, studies demonstrate IP prevalence’s of up to 50% [16–21]. Some studies have also found IP to occur more frequently in women [13, 22]. In medical students, IP has been associated with feelings of shame and inadequacy [23], stress [13, 20], anxiety [16, 18], depression [16], burnout [16, 17], and suicidal ideation [24].

While many studies have focused on factors that can contribute to the high burden of mental ill health among medical students, fewer studies have looked at ways to prevent or resist its development. However, to counteract psychological distress – knowledge of protective factors, such as resilience, is equally important. A shared definition within the higher education context is lacking [11], but resilience can be described as a person’s ability to bounce back and recover when facing stress and adversity [25]. Previous studies have found that active or approach-oriented coping strategies such as seeking social support, reflection, and problem-solving are positively associated with resilience to mental health issues among medical students – as opposed to so-called avoidance-oriented coping strategies, such as avoiding anxiety-arousing stimuli [26–29]. Higher resilience has been associated with, e.g., well-being, lower levels of distress and depressive symptoms, and a more favorable impression of the learning environment within the medical student population [30–32].

Medicine is laden with stress and difficulties, and making mistakes are inevitable. It is reasonable to believe that many of the feelings and behaviors commonly found among those with IP, such as, e.g., focusing on mistakes and pushing oneself to the limit to prevent failure [14] makes it difficult to bounce back from and cope with stress and difficulties. Researchers have also recently suggested that resilience could be improved by combating IP [33, 34]. Still, the possible correlation between IP and resilience among medical students remains largely unexplored. So far, only one cross-sectional questionnaire study has investigated the correlation between IP and resilience in medical students, in which the authors concluded that high levels of resilience likely protect against IP [35].

In Sweden, like other countries, the health of medical students is of concern for medical education institutions, and there is a need to understand the determinants of medical students’ well-being and help them manage the challenging academic and clinical environment and be proactive in preventing mental health issues. Research conducted across multiple nations, professions, and student groups suggests that IP is not unique to any context [36]. Thus, one can assume that neither medical students in Sweden are spared from this threat against mental health. However, to our knowledge, no published study reports the prevalence of IP nor its correlation to resilience among Swedish medical students.

Given the high levels of psychological distress reported among medical students and the known association between IP and mental illness, we need to advance our understanding of this phenomenon. Furthermore, more knowledge of protective factors like resilience is vital in designing evidence-based interventions to mitigate psychological distress during medical school. Therefore, this study aimed to investigate the prevalence of IP and its association with resilience among undergraduate medical students. Furthermore, we assessed differences between subgroups of students regarding IP and resilience, focusing on gender, age, and attending semester.

## Methods

### Study design and setting

This study is part of a larger project exploring students’ well-being at Umeå University, Sweden.

We chose a cross-sectional study design to explore the prevalence of IP and its association with resilience among students attending medical school at Umeå University, as no previous Swedish study has been conducted on this topic.

Undergraduate medical education at Umeå University – and in Sweden – is changing from a 5.5-year program, followed by an 18- to 24-month internship, to a 6-year program that grants students a medical license. Semesters 1 to 5 are pre-clinical, while 6 to 11/12 are clinical. At the time of this study, some participating students were still in the 5.5-year medical program.

### Measures

We created a web-based questionnaire using Microsoft Forms containing sociodemographic questions assessing students’ semester, age group, and gender and scales measuring IP and resilience. Both scales were validated and professionally translated from English to Swedish. Since the CIPS had not been translated into Swedish before, a translation from English to Swedish was first made, followed by a back translation to English. Two independent professional translators made the translations. Andra Gallis and Pauline Clance then made further retouches before the Swedish version of CIPS was finally approved.

To detect the frequency of IP feelings, the Clance Impostor Phenomenon Scale (CIPS) questionnaire was used [37]. The validated CIPS consists of 20 items that assess traits such as fear of assessment, fear of being less capable than others, and fear of being unable to repeat success [14, 38]. The items are scored on a 5-point Likert scale ranging from 1 to 5 (1 = not at all true and 5 = very true), reflecting the degree of impostor feelings, ranging from 20 to 100. A total score of 62 was used as a cut-off value to distinguish impostors from non-impostors [39]. The total CIPS score was also divided into four subgroups reflecting the degree of impostor feelings where 20–40 = Low, 41–60 = Moderate, 61–80 = Frequent, and 81–100 = Intense IP [37]. Approval to use the CIPS was obtained from Dr. Pauline Rose Clance.

The level of resilience was assessed using the Brief Resilience Scale (BRS) [25, 40] since it is recommended when studying resilience at a basic level, i.e., focusing solely on one’s ability to bounce back from adversity [41, 42]. The BRS consists of six statements, e.g., ‘I usually come through difficult times with little trouble’ and ‘I tend to bounce back quickly after hard times’, each with five alternatives indicating the degree of agreement. The total points of the BRS are then calculated, and the points are divided by statements answered. A score of 1.00-2.99 denotes low, 3.00-4.30 normal, and 4.31-5.00 high resilience [40].

## Data collection and participants

During February and March of 2023, all medical students registered in semesters 2, 3, 4, 5, 6, 7, 8, 9, and 10 at Umeå University (*N* = 944) were invited to participate in the study via the learning management system Canvas.

The invitation contained information about the study – including that participation was anonymous and voluntary – and a link to the online questionnaire. Three reminders to fill in the questionnaire were sent to each semester.

Of the 944 invited medical students, 457 responded, resulting in a response rate of 48%. Among the participants, 62.6% identified as women, 36.1% as men, and 1.3% as others. The largest age group was under 24, comprising 57.2% of the participants. Participants’ demographic characteristics are presented in Table 1.

**Table 1:** Demographic characteristics of the participants and mean scores for CIPS and BRS

	Number of participants (%)	CIPS (± SD)	BRS (± SD)
Total	457 (100.0%)	64.39 ± 17.45	3.41 ± 0.89
Attending semester			
2	52 (11.4%)	61.94 ± 16.82	3.42 ± 0.88
3	53 (11.6%)	66.89 ± 14.70	3.36 ± 0.99
4	50 (10.9%)	64.34 ± 17.60	3.55 ± 0.84
5	50 (10.9%)	64.14 ± 19.25	3.33 ± 0.83
6	51 (11.2%)	64.51 ± 17.16	3.36 ± 0.93
7	44 (9.6%)	63.66 ± 17.72	3.41 ± 0.87
8	50 (10.9%)	63.84 ± 18.82	3.44 ± 0.91
9	54 (11.8%)	66.74 ± 18.88	3.14 ± 0.94
10	53 (11.6%)	63.21 ± 16.60	3.69 ± 0.79
Age			
≤ 24	262 (57.2%)	65.06 ± 17.37	3.30 ± 0.89
25–29	129 (28.4%)	62.74 ± 17.45	3.55 ± 0.92
30–34	46 (10.0%)	67.43 ± 16.29	3.53 ± 0.87
≥ 35	20 (4.4%)	59.25 ± 16.29	3.69 ± 4.72
Gender			
Women	286 (62.6%)	67.36 ± 17.27	3.27 ± 0.87
Men	165 (36.1%)	59.34 ± 16.60	3.67 ± 0.88
Other	6 (1.3%)	61.67 ± 19.62	2.89 ± 1.01

### Statistical analyses

From Microsoft Forms, data from the completed questionnaires were exported to Microsoft Excel and subsequently imported into the software IBM SPSS Statistics 28 for analysis. All questionnaires in the datasets were complete, without any missing values for any variable. All data were normally distributed.

Total scores for CIPS and BRS were calculated for each participant, and comparisons were made between the demographic groups. Separate one-way ANOVA analyses were conducted to compare CIPS and BRS scores across age groups. Similarly, separate one-way ANOVA analyses were conducted to compare CIPS and BRS scores across semester groups.

Due to small sample sizes in higher age groups, participants were recoded into a new group labeled *≥* 35 years for statistical purposes. Post-hoc pairwise analyses were conducted using the Tukey HSD tests to determine if there were significant differences between groups. The independent sample t-test was employed to compare the scores for CIPS and BRS between men and women. The six participants who defined themselves as ‘other’ were excluded from the analysis due to the small sample size. Pearson’s correlations were used to investigate the relationship between CIPS and BRS scores. The level of statistical significance was set at *p* ≤ .05.

### Ethical considerations

The participants in the study received information about the study and consented to participate by completing the questionnaire. Since participation was voluntary, the questionnaire anonymous, and no personal data was gathered, the study was considered exempt from requirements for approval by the Swedish Ethical Review Authority.

## Results

### Comparison of Clance Impostor Phenomenon Scale and brief resilience scale with demographic characteristics

Among participants, the overall mean scores for CIPS were 64.39 (SD ± 17.45), and for BRS, the mean was 3.41 (SD ± 0.89). Participants’ demographic characteristics and mean scores for CIPS and BRS are presented in Table 1.

An independent t-test showed that women had significantly higher scores on CIPS and lower scores on BRS compared to men t(449) = 4.82, *p* = < 0.001 and t(449) = − 4.67, *p* = < 0.001, respectively. The findings suggest a medium effect size (Cohen’s d of 0.47 and − 0.46 for CIPS and BRS, respectively).

A one-way ANOVA showed that attending semester did not have any significant impact on CIPS scores, F(8, 448) = 0.43, *p* = .903. Similarly, a one-way ANOVA showed that age groups did not have any significant effect on CIPS scores, F(3, 453) = 1.57, *p* = .196.

For BRS scores, a one-way ANOVA showed that attending semester did not significantly affected scores, F(8, 448) = 1.52, *p* = .148. In contrast, a one-way ANOVA revealed a significant overall difference in BRS scores across age groups (F(3, 453) = 3.18, *p* = .024), with higher age groups scoring higher – see Table 1. However, post hoc analyses did not identify significant differences between age groups.

### Distribution of subgroups for Clance Impostor Phenomenon Scale and brief resilience scale

Of all participants, 58.4% scored above the CIPS cut-off value for experiencing IP. Further categorization of CIPS according to the CIPS scoring guidelines showed that 10.3% of participants had low feelings of IP, 29.5% moderate, 41.6% frequent, and 18.6% intense. Of all participants, 89.7% experienced at least moderate and 60.2% frequent to intense IP levels. The distribution of participants between different CIPS subgroups is presented in Table 2.

**Table 2:** Distribution of participants among different CIPS subgroups

CIPS subgroup	Number of participants	Percentage (%)
Low	47	10.3%
Moderate	135	29.5%
Frequent	190	41.6%
Intense	85	18.6%

When the BRS scores were categorized to investigate the level of resilience, the largest proportion of participants (53.1%) were categorized as having normal resilience. The distribution of participants between different BRS subgroups is presented in Table 3.

**Table 3:** Distribution of participants among different BRS subgroups

BRS subgroup	Number of participants	Percentage (%)
Low resilience	128	27.9%
Normal resilience	243	53.1%
High resilience	87	19.0%

### Correlation between Clance Impostor Phenomenon Scale and brief resilience scale

**Fig. 1:**
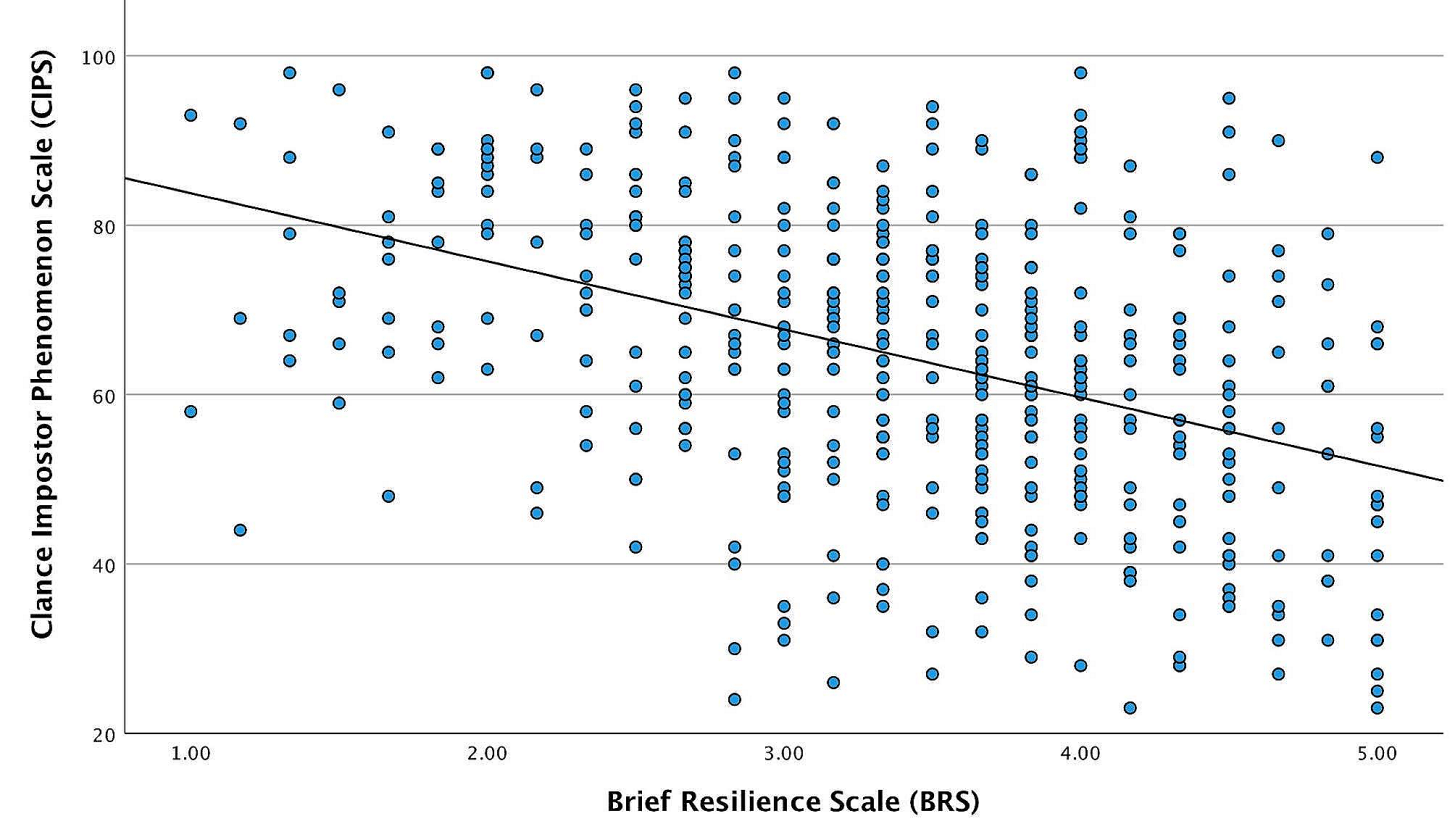
Correlation between impostor phenomenon and resilience among medicals students (*n* = 457). Pearson correlation coefficient (ρ=−0.412; *p*-value = < 0.001)

Correlation analysis showed a significant negative association between BRS and CIPS scores (*r* = -.412, *p* < .001) – see Fig. 1.

## Discussion

This is the first study to examine the prevalence of IP and its relationship with resilience among undergraduate Swedish medical students. We found that irrespective of age group and semester, more than half of all participants scored above the cut-off value indicating IP – and of these, most were categorized within the subgroup frequent IP-feelings. When looking at BRS scores, more than half of all participants had normal resilience, and which semester students attended did not significantly affect these scores. However, there was a significant overall difference across age groups, with those in the older groups scoring higher. Furthermore, female students had significantly higher CIPS and lower BRS scores than men. Lastly, the results showed that resilience had an inverse relationship with IP. The results from this study offer important insights into IP and resilience among medical students to inform possible curricular changes aimed at strengthening their well-being and expand upon previous work regarding the association found between IP and resilience.

### Do most medical students feel like an impostor?

Our results demonstrated a considerable prevalence of IP, indicating that the majority of Swedish medical student feels like an impostor – findings resembling those in previous studies [17, 19, 20]. Our results further showed that, in line with some earlier studies, the high frequency of IP remained stable across all studied semesters [16, 20, 21, 24]. This finding could indicate that IP is ubiquitous in this population before they begin medical school. Another possible explanation could be that the students have gone through high school as the best or among the best in their class, and when they start medical training, they are surrounded by high-achieving individuals, causing a rise in IP feelings. Our cross-sectional study design precludes any conclusions on these matters. Still, the even level of IP seen between the different semesters adds to previous studies, which have shown conflicting results regarding whether IP falls, rises, or remains stable throughout medical education. In some studies, the prevalence was lower in higher semesters, indicating that students learn to cope with the stressors in medical school. [13, 43] Other studies have found that the proportion of students with high levels of IP increased as their education progressed, suggesting that, e.g., transitioning from pre- to clinical training might constitute a risk for increasing IP feelings [17, 20, 44]. Even if the results from previous studies give an inconclusive picture, they indicate that IP can change during the education – for the better or the worse. However, it should also be noted that, like ours, all but two of the studies mentioned above [20, 44] were cross-sectional, making it difficult to draw any conclusions on causality. Additionally, we did not see any notable differences in IP between age groups. Previous studies, in contrast, have shown that the CIPS score tends to fall with increasing age [43]. In our study, however, we merely examined age groups, and it is possible that if we had known the students’ exact age, we would have also found a difference.

With respect to resilience, our results showed that more than half of all participants had normal levels – as seen in previous studies [3, 45] – which semester students attended did not significantly affect these scores. Still, almost one-third of our participants displayed low resilience, suggesting that many students may struggle to maintain their psychological well-being. Furthermore, another study that measured medical students’ resilience at five points during the academic year, found that although most students exhibited normal resilience, their BRS scores decreased throughout the year [3] – indicating that resilience is a perishable commodity that can quickly fall. Therefore, even students who initially display normal levels are at risk of running short of this protector against stress and adversity. Additionally, we observed that students in older age groups tended to score higher on the resilience scale, although the differences between groups did not reach statistical significance. It has been suggested that the positive association between resilience and higher age in previous studies can be explained by the fact that with age comes more life experience through which people acquire coping strategies and resources needed to build resilience [46].

Our results also demonstrated an inverse relationship between IP and resilience, indicating that IP could be a barrier to resilience – or vice versa – especially among female students. As suggested by other scholars, a high frequency of IP feelings could hinder students from reaching higher resilience [33, 34, 47]. The tendency to focus on one’s mistakes and fear of not being good enough associated with IP-feelings [14] likely cause a vicious cycle of striving for perfection and pushing oneself to the limit to prevent failure, effectively counteracting the ability to bounce back from the stress and adversities that are inevitable parts of medicine. In addition, this likely leads to difficulty in seeking support from others – a coping strategy suggested to be essential for developing resilience [26–29]. Likewise, low resilience *could* exacerbate the harmful effects of IP. For instance, students with low resilience might struggle more to manage the emotional burden of IP, leading to increased stress. Our cross-sectional study design, though, means no temporal relationship can be established. Nevertheless, based on previous studies, it is safe to say that impostor feelings *do* contribute to psychological distress [13, 16–18, 20, 23, 24], while high resilience *does* protect against stress and mental ill health [30–32] – putting students displaying both high IP and low resilience at increased risk of adverse mental health. Thus, longitudinal studies are needed to explore the causal relationship between IP and resilience among medical students.

### Are female medical students at higher risk for IP and low resilience?

Regarding gender, our results showed that female medical students had significantly higher CIPS scores and lower BRS scores than male students. Other studies have also found higher IP [3, 12, 16, 17, 22, 43] and lower resilience [3, 45, 48] among female than male medical students, although these results are not unanimous. This study does not answer why our female participants had higher IP and lower resilience than their male counterparts. Still, this troublesome finding cannot be left uncommented.

It is reasonable to believe that the gender bias and inequality shown in medicine, which negatively impact female students, interconnect with IP and resilience [49]. Many studies – also from Sweden and Umeå – have shown that female and male medical students have different experiences during medical education. Stereotypical notions that the physician is a man cause female students to be mistaken for nurses and made invisible, undermining their opportunities to be taken seriously as physicians-to-be [50]. In addition, female medical students are often subjected to discrimination and sexual harassment [51–53]. Furthermore, from their dependent position, students often have to cope with these injustices by, e.g., working hard to prove themselves capable and playing on gender stereotypes [50]. These strategies have much in common with the behaviors Clance and Imes [18, 54] have described as contributing to sustaining IP, e.g., diligence, hard work, and using charm and perceptiveness to win the approval of superiors.

However, when talking about IP, there is a trend in the current literature of attributing the phenomenon to personal characteristics, like perfectionism [55]. In their commentary, “Impostor Syndrome, treat the cause not the symptom,” Mullangi and Jagsi [55] rightfully point out that this entails a risk of drawing attention *from* the prevailing systemic problems with discrimination and sexual harassment and *to* something which is presented as more of personal challenge. Furthermore, this shift in perspective suggests that to mitigate the high levels of IP, we should focus on “fixing” affected individuals rather than addressing the structural environment. However, as suggested by Mullangi and Jagsi, IP can and should be viewed less as a personal challenge and more as a large-scale systemic problem with detrimental consequences for those affected – calling for a need to address this problem at an organizational level. At this point, however, with the extensive levels of IP and the psychological distress and suffering that comes with it, perhaps we have no choice but to treat both the cause (inequity) and the symptoms (IP). As many men are affected by IP as well, the best strategy to mitigate IP and its negative consequences is probably to implement prevention efforts on organizational/institutional, group, and individual levels – an approach supported by a recent scoping review on educational interventions for IP in health care [56].

### Medical education implications

Considering the high levels of frequent to intense IP found among our participants and its known association with adverse health outcomes, interventions aimed at mitigating IP among medical students should be prioritized for medical educators. Previously suggested strategies to alleviate IP include e.g., seeking recurring feedback from trusted people [14] and practicing self-compassion [34, 57]. Recent studies on health care professionals and other student groups have also shown promising results from impostor-reducing interventions using, e.g., taped confessions from individuals describing their own experiences of IP, information on specific coping strategies to address IP [58], and interactive discussions on the topic [33]. Similar longitudinal interventions addressing IP should be performed among medical students. Based on our results, showing that medical students pretty much enter medical school feeling like frauds already without any alleviation in higher semesters, such interventions should be introduced early in the curriculum and as described above, be directed on multiple levels. We suggest that on a group level, all medical school classes should receive lectures on IP – including its causes and strategies that can be used to handle it – combined with small group discussions and exercises where students are encouraged to reflect on these matters. For those students suffering from intense levels of IP, we also suggest individual-oriented interventions to overcome these feelings. For example, exercises practicing self-compassion and mindfulness, stress management courses, and structured feedback from mentors.

Although most of our participants possessed normal levels of resilience and thus were equipped with basic psychological resources to cope with challenges throughout medical school, this may not be enough. Given the high levels of stress that medical education and the medical profession entail, it would be beneficial for medical students to obtain high(er) resilience to prevent and address psychological distress. Thus, strengthening resilience should also be a priority for medical education. According to a review on fostering resilience among healthcare students, previous studies have evaluated several interventions, such as cognitive-behavioral therapy, mindfulness training, and stress inoculation [59]. The authors concluded that although their review suggests positive effects of resilience training, better study designs, e.g., consensus on the definition of resilience and longer follow-up times, are needed to draw certain conclusions. Based on previous studies showing that approach-oriented rather than avoidance-oriented coping strategies are positively associated with resilience [26–29], we would also like to propose intervention studies exploring whether educational interventions could successfully support students in coping with adversity and evaluate whether this can strengthen their resilience. Considering the inverse relationship between IP and resilience seen in our results, we also suggest that a comprehensive approach should be considered for future studies exploring whether counteracting IP could strengthen resilience – and vice versa.

Concerning interventions to strengthen medical students’ well-being, however, combating IP and promoting individual resilience may be insufficient [60]. Even though we should teach students about IP and resilience, this must be accompanied by understanding the root causes of well-being – and lack thereof. The problem does not usually lie within the individual medical students – it likely lies mainly within the medical culture. Thus, more interventions and studies are also needed to investigate how aspects of the medical culture, such as gender stereotypes, perfectionism [24, 61], and stigmatization of mental health issues [26, 62, 63], may impact IP and resilience – studies that go beyond individual factors and address structural barriers to well-being. Such interventions could, for example, be focused on medical school faculty and physicians, encouraging them to act as role models for medical students by acknowledging the stressful nature of medicine and discussing the fact that mistakes, and set backs are inevitable parts of medicine. Furthermore, both students and faculty need education on gender bias. Such institutional initiatives should be evaluated in longitudinal studies to determine if they were efficient in changing the medical culture and in mitigating IP as well as promoting active coping strategies and strengthening resilience.

### Strengths and limitations

The results of this study shed light on IP and resilience among medical students. However, it is crucial to consider its limitations when interpreting the findings. First, the respondents may not be representative of the entire medical student body at Umeå University. For example, there is a risk of selection bias where individuals who experience more IP feelings prioritize participation, affecting the mean CIPS score. Furthermore, the first and last semesters were not represented among the participants, which may alter the mean age and CIPS and BRS scores. Second, the cross-sectional study design means that no causal conclusions can be made. As described previously, addressing this limitation, a longitudinal intervention focusing on strengthening resilience and measuring resilience and IP could be implemented while mitigating the high IP levels among medical students. Third, our decision to use the brief BRS scale, which only measures the ability to recover from stress [25], means that we cannot say anything about individual students’ characteristics that could promote adaptation to stress. Therefore, future studies are needed based on scales assessing resources that may promote resilience. Fourth, we did not explore the situation for discriminated social groups such as underrepresented ethnic minorities. Previous studies have shown that exposure to stereotype threat and discrimination make minority groups more susceptible to IP [64, 65]. Hence, to effectively mitigate IP, future studies should explore this important aspect further. Finally, the study was conducted at a single medical school, which could limit the generalizability of our results. Nevertheless, despite the described limitations, the study design allowed us to explore a large number of students (*N* = 457) and present the first CIPS and BRS data in Swedish medical students, enabling comparisons in future studies between different student populations. For example, between students in different cultures/countries and between medical students studying in their birth country and those studying medicine abroad – which is common among Swedish medical students/physicians.

## Conclusions

This study explored the prevalence of IP and its association with resilience among Swedish medical students. It suggests that the majority of medical student feels like an impostor – and many of these students suffer from severe IP. In addition, our results imply that women have higher levels of IP and lower levels of resilience than their male co-students. Lastly, our results showed that resilience was inversely associated with IP, implying that higher levels of resilience could protect against IP. Future longitudinal studies must explore how gender might contribute to IP feelings and investigate the potential advantages of reducing IP and strengthening resilience among medical students through curricular interventions. However, targeted interventions that only address individual students’ IP and resilience are probably insufficient to enhance medical students’ well-being. There is also a need to address aspects of the medical education culture that may contribute to IP, such as gender stereotypes and norms on perfectionism.

## Data Availability

The datasets generated during this study are available from the corresponding author on reasonable request.

## Abbreviations

IP: Impostor Phenomenon
CIPS: Clance Impostor Phenomenon Scale
BRS: Brief Resilience Scale
